# Identifying the determinants of non-injection of covid-19 vaccine: A qualitative study in Urmia, Iran

**DOI:** 10.3389/fpubh.2022.927400

**Published:** 2022-08-04

**Authors:** Javad Yoosefi Lebni, Seyed Fahim Irandoost, Sardar Sedighi, Sina Ahmadi, Rana Hosseini

**Affiliations:** ^1^Social Determinants of Health Research Center, Lorestan University of Medical Sciences, Khorramabad, Iran; ^2^Social Determinants of Health Research Center, Clinical Research Institute, Urmia University of Medical Sciences, Urmia, Iran; ^3^Faculty of Physical Education, Sports Management Group, Tabriz University, Tabriz, Iran; ^4^Social Determinants of Health Research Center, University of Social Welfare and Rehabilitation Sciences, Tehran, Iran

**Keywords:** determinants, COVID-19, vaccine, non-injection, qualitative study

## Abstract

**Objective:**

Extensive vaccination coverage is one of the most effective ways to control COVID-19 vaccine, but the tendency to inject the vaccine is always hampered and there are various determinants of non-injection. Hence, the present study was done with the aim of identifying the determinants of non-injection of COVID-19 vaccine with a qualitative approach in the city of Urmia in Iran.

**Methods:**

The present study was conducted with a qualitative approach and conventional content analysis method among 36 people who refused to be vaccinated. Access to participants and data collection was done in person (28 interviews) and online (8 interviews) through targeted sampling and snowball method and semi-structured interviews. Data management was performed using MAXQDA-2018 software and its analysis was performed by Graneheim and Lundman method. Also, Guba and Lincoln criteria were observed to improve the quality of results.

**Results:**

After analyzing the data, 3 main categories and 11 subcategories were obtained including (1) Individual factors (fear of short-term side effects of vaccine, personality traits, distrust of vaccines and pharmaceutical companies), (2) Socio-cultural factors (conspiracy theory, social learning, misconceptions about COVID-19, fatalism), legal and managerial factors (incomplete information, difficult and irregular access to vaccination centers, lack of restrictions and compulsion to be vaccinated, lack of incentives to be vaccinated).

**Conclusion:**

The results showed that various determinants were involved in the non-injection of COVID-19 vaccine. Therefore, efforts to increase vaccination coverage require comprehensive measures at different levels and cross-sectoral cooperation between governmental and non-governmental institutions and organizations.

## Introduction

With the outbreak of COVID-19 from China in 2019 and the global involvement with it, tackling and preventing it has become a major challenge worldwide (1–4). The incidence and mortality of this disease has reached more than 448 million people infected and more than 6 million deaths. The highest number of cases in the world belongs to the United States and Iran has recorded more than 7 million infected and 138 thousand deaths (5).

One of the most effective ways to control high rates of COVID-19 infection is widespread vaccination coverage worldwide (6). Once vaccine is discovered and available, its acceptance by individuals is a key issue in controlling the disease, as high public coverage of vaccine injection is required to maintain human health (7, 8). In fact, more the establishment of collective immunity, the major part of the population must be vaccinated (9). Contrary to what research shows about the tendency of most people in getting vaccinated during epidemics (10), with the start of COVID-19 vaccination, there was no high willingness to inject (11). Recent public opinion polls also show a decline in skepticism about vaccination; In the United States, for example, 20 to 27 percent of people report refusing to be vaccinated against the virus (12, 13). In a the study in Iran, about 62% of the respondents intended to be vaccinated with COVID-19, while 38% were hesitant or completely opposed to the vaccine (14).

There are different reasons for unwillingness to be vaccinated (10, 11, 15, 16) and it is important to understand the factors that make vaccination doubtful (6). A study in Iran showed that, age, fatalism, and socioeconomic status had significant associations with the COVID-19 vaccine acceptance (17). According to research on risk perception and behavior, those who do not have a favorable attitude toward COVID-19 vaccination find the virus less threatening (11). The intention to be vaccinated is also included under the influence of cognitive assessments (probability of outcome) (18), race and ethnicity (19–21), perceptions of risk and susceptibility to COVID-19 (22), ease of availability and access (23, 24) and socio-economic and socio-demographic status (15, 18, 22).

There are lots of reason to reject, delay, hesitancy and refusal of COVID-19 vaccine: Fear of short or long term applications to health, not considering being in a risk group, pregnancy, risk pregnancy, recent abortion, breastfeeding period (25), misinformation beliefs (26, 27), not having enough vaccine-related information (26–28), concerns over vaccine safety (18, 19, 26, 28, 29), structural barriers (28, 30), personal experience with the disease (31, 32), possible unknown future adverse effects of the vaccine (32, 33), social determinants of health (23), low health literacy (23, 34), not trusting the drug companies (23, 27, 29), political ideologies (11, 23, 35), distrust of science and the government (19, 26, 29, 33). Also, various social media made people hesitate to inject the vaccine by spreading false rumors about the COVID-19 vaccine harms, which could create a major challenge for the health system (36, 37). A study in Germany showed that health concerns, Low perceived benefit of vaccination, lack of information, systemic mistrust low perceived risk of contracting COVID-19 and spiritual or religious reasons were reasons for refusing a COVID-19 vaccination (16).The results of a study showed that believing in the illusion of a vaccine plot, the perceived severity of COVID-19, being a man, household income, not paying attention to conservative parties, and not relying on social media for gaining information about the virus are reasons to willingness to vaccine injection and concerns about vaccine efficacy, individual susceptibility and severity of COVID-19, and the possibility of immunity from previous COVID-19 infection were among the reasons for unwillingness to be vaccinated (9). In the another study, concerns about the side effects and safety of the COVID-19 vaccine, distrust to the government, and concerns that the COVID-19 vaccine was made too quickly were the main reasons for delaying vaccination (38).

Given the importance of vaccination for collective immunity and the control of COVID-19 disease, it is important to identify the reasons why people refuse to be vaccinated. This importance is highlighted by the emergence of COVID-19 and the lack of research in the field of identifying the determinants of non-vaccination. Another aspect of the importance of the present research is that search and examining of the researchers have shown that no study is done in Iran to identify the reason of unwillingness to vaccination and the few studies performed, have examined the predictors of vaccination intent (6, 8). In general, although quantitative studies have been conducted to investigate the reasons for the intention to COVID-19 vaccination in Iran and the world, but few studies have examined the reasons for non-vaccination from a qualitative and comprehensive perspective. Therefore, the present study was performed with the aim of identifying determinants of non-injection of COVID-19 vaccine with a qualitative approach in Urmia, Iran.

## Methods

### Design

The present study was conducted with a qualitative approach and conventional content analysis method in the city of Urmia in Iran in 2022. Since the purpose of the research is more to understand the phenomenon than to predict, and also due to the complexity of the subject under study and the anonymity of samples, the researchers used a qualitative approach and conventional content analysis method for this research. Content analysis is a systematic approach that can be used to discover large amounts of textual information by coding and classifying data in order to recognize the process and patterns of words, their relationships, structures and communication discourses (39). Urmia as one of the metropolises of Iran, the center of West Azerbaijan province, is located in the northwestern of Iran and it is the 10th most populated city in Iran and the second most populous city in the northwestern region of Iran according to the 2016 census with 736,224 population. Urmia has the first modern hospital and the first modern medical education center in Iran, either (40).

### Participants

Participants in the research were those who met the conditions set by the Iranian Ministry of Health to receive the COVID-19 vaccine but refused to be vaccinated. Inclusion criteria were meeting the conditions to inject COVID-19 vaccine and prevention of injection (ordinary opposing people not anti-vaccine groups), being over 18 years old, willingness to participate in the study and observing health protocols during the interview. Exclusion criteria also included belonging to anti-vaccine groups and the preachers of non-vaccination, discontinuation of the interview and unwillingness to participate until the end of the interview process and being under 18 years of age. Thus, people who were in the anti-vaccine groups were excluded and normal people in the community who were not vaccinated were included in the study.

### Data collection

Participants were selected based on purposive sampling and snowballs. By referring to the city and asking people, as well as using the information available in health centers, individuals who refused to receive the COVID-19 vaccine were identified. At the end of the interview with these people, they were asked to introduce other people who meet the inclusion criteria to the researchers. The main method of data collection was semi-structured face-to-face interview (28 people), but in interviews with 8 participants, at their request, telephone and online interviews (using WhatsApp software) were used. With the consent of the participants, the whole interview process was recorded for coding and analysis, and if necessary, the researcher also used field notes. Interview questions were developed after holding two sessions between the research team and also conducting three pilot interviews (Table 1). The interviews were conducted by the third author of the article and under the supervision of the first and second authors. In the interview process, first the researcher introduced himself and then briefly explained the research necessity to the participants and by receiving written informed consent from them, the interview started with a few demographic questions and continued with the main questions. The duration of the interviews varied according to the information provided by the participants, so that the minimum interview was 25 min and the maximum was 120 min and the average was 63 min. The place and time of the interviews were determined by the participants, but an attempt was made to hold them in a secluded place without the presence of anyone else. Most interviews took place in public parks or in participants' private homes. Data collection continued until saturation, which was done in interview with the participant No. 29, but due to the sensitivity of the researchers and more confidence, 7 more interviews were conducted, which resulted in a total of 36 interviews. Theoretical saturation means when the continuation of the interviews does not add anything new to the research and the content and codes are repeated (41).

**Table 1:** Interview question guide.

**No**.	**Questions**
1	How much do you think the vaccine can protect the body against
	COVID-19? Explain.
2	Why not get vaccinated even though you had the vaccination conditions?
3	What were your main obstacles to getting the vaccine?
4	What do you think about the vaccines that are available? Explain.
5	How do you evaluate the activities of the health groups and the
	vaccination process? Explain.
6	Were people involved in preventing you from injecting the vaccine?
	Explain.

### Data analysis

The data classification and adjustment process was performed in MAXQDA-2018 software and its analysis was performed using Graneheim and Lundman method (42) by the first, correspond and second authors of the article. In the first step, the researcher typed the interviews in Word 2017 software immediately after the first interview and on the same day with the help of another research colleague. In the second step, the text of the interviews was read carefully by the researchers three times to get a general understanding of the text. In the third step, all the texts of the interviews were read line by line and word by word with great care and patience and the initial codes were extracted. In the fourth step, the codes that were similar in meaning and concept and could be in a category were placed in a category and how they were related was determined. The codes and categories were then placed in the main categories, which were more conceptually comprehensive and abstract, and the themes were extracted. Finally, in a joint meeting, the entire data analysis process was shared and the opinions of all research colleagues were used.

### Trustworthiness

Thirty two items of qualitative research report were observed (COREQ) (43). Guba and Lincoln criteria (44) were observed to improve the quality of results. To increase the credibility of the research, the researchers observed the principle of diversity in sampling and selected participants who were different in terms of demographic characteristics such as age, sex, etc. At the end of the interview, the researcher's general understanding of the participants' statements was briefly stated and confirmed. At the end of the data coding and analysis process, a table of categories, subcategories and codes with quotes was provided to 10 participants to determine whether the researchers had reported their experiences correctly or not which was approved by all the 10 participants. To gain Confirmability, the researchers sent the analyzed data and findings to four leading qualitative research researchers, as well as five health network staff and managers, and modifications were performed according to their opinions as needed. To increase the dependability, all project partners were informed of the analysis and coding process and expressed their views in the meetings that were held, and finally the names of the categories and subcategories were finalized with the approval of all authors. To increase transferability, a complete description of the entire research process was provided. The quotes of the participants were given directly and in large numbers and the research findings were sent to 9 people who met the inclusion criteria in this study but did not participate in the study and were approved.

### Ethical considerations

To observe the ethics principles of the study, all participants were given informed written consent, told that there was no obligation to participate in the study, and that they could discontinue the interview whenever they wished. They were also briefed on the interview process and how to publish the results, and were assured that their names would remain confidential when publishing the results. Also, during the face-to-face interview, all health protocols (such as using masks and gloves and observing the appropriate physical distance, air conditioning, etc.) were observed.

## Results

This study was conducted with the participation of 36 people whose demographic characteristics are listed in Table 2. Also, from the data analysis, 3 categories, 11 subcategories, and 97 primary codes were formed (Table 3).

**Table 2:** Demographic information of participants.

**Variables**	**Groups**	**Frequency**
Gender	Female	19
	Male	17
Marital status	Married	20
	Single	9
	Divorced/Widow	7
Education	Under diploma	15
	Diploma and associate's degree	13
	bachelor's degree and higher	8
Age	<40	7
	40–60	13
	>60	16
History of underlying disease	Yes	21
	No	15
History of getting COVID-19	Yes	12
	No	24

**Table 3:** Categories, subcategories, and codes obtained from interviews.

**Codes**	**Subcategory**	**Category**
Fear of fever and chills, fear of dizziness and nausea, fear of body aches, fear of blood clots, decreased immunity to the vaccine	Fear of short-term side effects of the vaccine	Individual factors
Laziness, lack of motivation, pride, lack of risk-taking, conservatism, carelessness about health	Personality characteristics	
Distrust in the development of immunity and long-term effectiveness of vaccines, distrust in foreign pharmaceutical companies, distrust in foreign vaccines, distrust in existing vaccines, variety of vaccines and persistence of infection and mortality despite vaccination	Distrust of vaccines and pharmaceutical companies	
Gene change, male sterilization, generational cleansing, autism, microchips entering the body to control humans, human origin of the virus and targeted vaccination, politicization of COVID-19 spread and vaccination	Conspiracy theory	Socio-cultural factors
No vaccination among friends and colleagues, no vaccination among relatives, no vaccination in some foreign countries, encouraging some celebrities and clerics not to get vaccinated	Social learning	
A person who has had a COVID-19 does not need a vaccine anymore, if I was supposed to get a COVID-19 I already had it, then I do not need a vaccine anymore, people only get a COVID-19 once, someone who is not old and has no underlying disease does not need a vaccine	COVID-19 misconceptions	
Not having a problem with COVID-19 death, knowing the COVID-19 origin as a divine destiny as well as its death, knowing disease and mortality to be natural	Fatalism	
Incomplete information about available vaccine types, Incomplete information about vaccine efficacy, Incomplete information about the need for COVID-19 vaccine	Incomplete information	Legal and managerial factors
Long queues for vaccines, lack of vaccination centers, poor information about vaccination appointments, difficult queuing process for vaccinations	Difficult and erratic access to vaccination centers	
No ban on entering public places without vaccination, no ban on entering specific places, no penalty for not being vaccinated	Lack of restrictions and compulsion to be vaccinated	
Failure to provide financial incentives for vaccination, failure to provide food purchase vouchers, non-provision of sports equipment vouchers, etc.	Lack of incentives to get vaccinated	

### Individual factors

The first category that emerged from the data analysis was individual factors, some of which pointed to fears of short-term effects of the vaccine, others to personality traits that prevented them from receiving the vaccine, and finally distrust of pharmaceutical companies and vaccines was an influential individual factor.

#### Fear of short-term side effects of the vaccine

Some participants stated that fear of short-term side effects of the vaccine, such as fever and chills, dizziness and nausea, body aches, and blood clots, prompted them to avoid the vaccine. In fact, most of these people saw such side effects in people close to them who had been vaccinated and therefore decided not to get vaccinated. Some other participants believed that the vaccine lowered the body's immunity, so they refused to inject it.

“*My father, who was vaccinated, was hospitalized for a week. He had a very bad fever and shivering. We were afraid he would die, so I regretted it and did not get vaccinated” (38-year-old woman, diploma)*

“*I heard that in some people the vaccine causes blood to clot in the brain and they may die, so I decided not to get vaccinated anymore” (54-year-old, below diploma)*

“*When you get vaccinated, your immunity goes down and you are more likely to get COVID-19 or other illnesses, which is why I chose not to get vaccinated” (61-year-old, below diploma)*

#### Personality characteristics

Having some personality traits such as laziness, lack of motivation, pride and lack of risk in some participants made them refuse to be vaccinated. Some people did not have enough time and patience to go to vaccination centers, and others felt so strong that they did not need to be vaccinated. In others, living conditions made them indifferent to their health and had no incentive to prolong life. Other participants were conservative and could not convince themselves of the risk of receiving the COVID-19 vaccine.

“*I have no happiness in this life to go get vaccinated and live longer. Whatever wants to happen, I will eventually die” (45-year-old woman, diploma)*

“*My body is so much stronger than that a COVID-19 can destroy it. I'm sure I will never take a COVID-19, and if I do, nothing will happen to me, so I no longer need to be vaccinated” (28 years old man, bachelor's degree)*

“*Nothing happens, I'm not too worried about getting a COVID-19” (55-year-old man, below diploma)*

“*I'm generally conservative so that I can't risk anything until everyone has done it, so I said not to get vaccinated until everyone has done it.” (33-year-old woman, Graduated)*

#### Distrust of vaccines and pharmaceutical companies

Most participants said they were unsure of the long-term effectiveness of vaccines. They also did not have much confidence in pharmaceutical companies, stating the companies were just looking for more wealth and power. Also, some other participants were distrustful of the foreign vaccines available in the Iranian market. A limited number of participants also criticized the illegality of importing the Pfizer vaccine in Iran, stating that if the Pfizer vaccine was present, they would change their minds and inject the vaccine. There were participants who said that despite the vaccine being given in the community, the death toll and high incidence still made them pessimistic about the vaccines.

“*I'm not sure if these vaccines can protect us against the COVID-19 forever, because it may mutate again and you may need to get another vaccine” (34-year-old woman, PhD)*

“*Pharmaceutical companies are only looking for their own interests. They are not looking for people's health. I cannot trust them and go to get the vaccine” (71-year-old, below diploma)*

“*Most of the vaccines they inject are foreign. I cannot believe them because they do not like us and our health may be endangered” (68-year-old woman, below diploma)*

“*I do not like any of these vaccines that are injected, so I did not go to get vaccinated. If it was Pfizer, I would go and get it” (40-year-old woman, graduated)*

### Socio-cultural factors

In addition to individual factors, there was a set of social and cultural factors that prevented people from receiving the vaccine. These social and cultural factors included conspiracy theories, social learning, misconceptions about COVID-19 and fatalism.

#### Conspiracy theory

In Iran, some people have rumored about vaccines that these vaccines seek to reduce the world's population or seek to purge generations by changing genes and sterilizing men. Others also believe that these vaccines continue to lead to autism and other diseases. Other participants believed that the purpose of vaccination was to inject microchips into the body to control humans, and believed that the virus was of human origin. They also considered the creation and spread of COVID-19 and vaccination to be generally political.

“*I did not go to get vaccinated because I believe that these vaccines are nothing but misery for people. I heard that they change genes and cause us a lot of problems” (37-year-old man, Associate's degree)*

“*I took my wife and got vaccinated, but I did not go myself because a friend of mine said that whoever gets these vaccines will become infertile” (48-year-old man, Associate's degree)*

“*By injecting the COVID-19 vaccine, things are inserted into the human body that can later be controlled or monitored later, so I was scared and did not go to get the vaccine” (74-year-old man, below diploma)*

“*In general, this COVID-19 was made by human hands and was produced for a specific purpose. The vaccine is already made for other purposes and neither the disease nor the vaccine cannot be trusted at all. Its vaccine may be contaminated with things that will later destroy our lives” (66-year-old woman, diploma)*

#### Social learning

Most participants were more than ever encouraged to do the same and refuse to get vaccinated when they saw people around them who had refused to be vaccinated. However, in Iran, some prominent people sometimes encouraged people not to receive the vaccine, and some people followed them. Also, the non-acceptance of COVID-19 vaccine in some foreign countries producing these vaccines was not ineffective.

“*When I saw that my brother did not get vaccinated, I did not get vaccinated anymore, since my brother reads a lot and he surely knows something that he doesn't vaccinated” (70-year-old man, below diploma)*

“*Some of my friends did not get vaccinated. I also doubted and did not go to get vaccinated” (42-year-old woman, bachelor's degree)*

“*I heard from some famous people that they said do not get vaccinated, they definitely have a reason for doing so. These things made me not get vaccinated” (34-year-old, PhD)*

#### COVID-19 misconceptions

In Iran, there were some misconceptions about COVID-19 that prevented people from getting vaccinated. Most of these beliefs stemmed from superstitions and poor health knowledge.

“*Anyone who has had a COVID-19 does not need to be vaccinated anymore. I got a COVID-19 once earlier. I no longer need to be vaccinated” (67-year-old woman, below diploma)*

“*People only get a COVID-19 once, and I got it once, I'm comfortable now” (70-year-old woman, diploma)*

“*Only older people need to get COVID-19 vaccine; it is not very dangerous for young people” (34-year-old woman, PhD)*

“*I'm not very old and I do not have a specific disease, so I do not need to get vaccinated anymore” (48-year-old man, diploma)*

#### Fatalism

Fatalism is another feature of Iranian society that made people believe in life and death in the hands of God and do not believe that the vaccine can extend their life. In fact, Iranian society is a religious and traditional society, which is why they know God as the source of many behaviors and events on earth and believes that nothing will happen without God's will. This is why they do not care much about their preventive behaviors in the face of COVID-19.

“*Death and life are in the hands of God. If God wills me to die, these vaccines can do nothing, and if God does not want me to die, if I get COVID-19 a thousand times, I still won't die” (67-year-old man, below diploma)*

“*It must have been destiny for COVID-19 to come, which is why we all have to accept it” (68-year-old woman, below diploma)*

### Legal and managerial factors

COVID-19 vaccination requires extensive coordination at all levels of society. Therefore, management and legal factors can play a decisive role, while in Iran these coordination did not always exist and managerial and legal factors such as incomplete information, difficult and irregular access to vaccination centers, lack of restrictions and compulsion to be vaccinated and lack of incentives to be vaccinated were the most important barriers to vaccination.

#### Incomplete information

Despite the variety of news, some people still do not have the necessary information about the COVID-19 and available vaccines. This incomplete information causes them to be skeptical about getting vaccinated. Some participants did not have enough knowledge about the types of vaccines available, their effectiveness, and the need for vaccines, which made it more difficult for them to make decisions.

“*I do not know exactly which vaccine is good and which is bad, and what is the difference between them? I was afraid to get one and then regret that I did, that's why I did not go to get the vaccine”*

“*I do not know exactly what each vaccine is better for” (73-year-old woman, below diploma)*

“*I heard that anyone who gets the vaccine can still get COVID-19, so in practice these vaccines are not very effective” (68-year-old woman, below diploma)*

#### Difficult and erratic access to vaccination centers

At the beginning of the vaccination process, the import of vaccines was very low and the vaccines made in Iran had not yet reached the injection stage. Of course, the small number of vaccination centers and the lack of a proper process for scheduling vaccinations were also effective. These issues made some people reluctant to go to vaccination centers.

“*Once I was passing by one of the vaccination centers, I saw a large crowd of people in line, so I totally let it alone” (73-year-old woman, below diploma)*

“*It was a long distance from my place of residence to the vaccination center. I could not go and no one was there to help” (70-year-old man, diploma)*

“*How to que wasn't clear exactly; anyone who went there was vaccinated without being queued” (37-year-old woman, Associate's degree)*

#### Lack of restrictions and compulsion to be vaccinated

In Iran, there were no restrictions and compulsion for those who refused to receive the vaccine, so some people had no reason to get vaccinated. Although some restrictions have recently been imposed on people who have not been vaccinated, there has been no monitoring.

“*I think there should be a series of restrictions for those who do not get vaccinated. For myself, if I was told my subsidy is to be eliminated if I do not get vaccinated, I would get vaccinate.” (43-year-old man, Diploma)*

“*I see no reason to get vaccinated and no one can force me to get vaccinated' (34-year-old woman, PhD)*

“*I only get vaccinated if there is a penalty for not doing it” (55-year-old woman, Associate's degree)*

#### Lack of incentives to get vaccinated

In Iran, there is no incentive for people to get vaccinated, while financial and non-financial incentives may increase acceptance.

“*If I was paid money or something, I would get vaccinated” (28-year-old man, Bachelor's degree)*

“*I think maybe even if very small incentives were considered for those who were vaccinated, people would be more accepting, but unfortunately there is nothing, so people are not in the mood to go and get vaccinated” (73-year-old woman, below diploma)*

“*Maybe if was given a voucher to buy food or exercise equipment or anything else, I would get vaccinated even if it was of low value” (34-year-old woman, PhD)*

## Discussion

The aim of this study was to identify the determinants of non-injection of COVID-19 vaccine with a qualitative approach. The results showed that there are reasons for not receiving the COVID-19 vaccine. These are discussed below.

Fear of short-term side effects of the vaccine was one of the reasons for not injecting the vaccine in the participants of the present study. Numerous studies have reported the fear of side effects and concern about the adverse effects of the vaccine plays a role in not injecting it in the population (32, 38, 45–47). The vaccine is always associated with side effects, and its injection has been a kind of phobia for humans, and this issue has been more pronounced in the case of COVID-19. It can be said that the reason for this fear of injection is due to the observation of side effects among those around and family, and because COVID-19 vaccination is performed in the general population, the observation of its side effects is more than the vaccines that are injected in certain groups.

Personality traits such as laziness, lack of risk-taking, knowing oneself as strong, and indifference to health were other determinants of not receiving the COVID-19 vaccine. Some studies showed that a negative attitude toward COVID-19 vaccination and personal and clinical characteristics makes a person consider it less threatening and does not attempt to inject the vaccine (11, 48). In general, people who are lazy in personality or consider themselves strong and believe that they do not need the vaccine need more interventions and training, because they do not believe in preventive measures and allow the disease to spread further.

Another determinant of non-vaccine injection in the present study was distrust of vaccines and pharmaceutical companies. Consistent with our findings, the results of various studies have shown that doubts about the effectiveness and safety of vaccines are among the reasons for not wanting to be vaccinated (9, 22, 29, 33, 48–53). In the study of Khankeh et al. (47) in Iran, there was insufficient social trust in health system officials and pharmaceutical and vaccine companies. In another studies also those who distrusted the government, delayed vaccination (22, 54). In general, the emergence of COVID-19 and the short time it takes to make a vaccine have made people skeptical about its function. At the same time, trust and social capital are key factors in complying with COVID-19 instructions and vaccination, which has more complex dimensions in Iran. In Iran, social capital is at a weak (low) level and the relationship and interaction of the government with the nation is not a two-way relationship based on cooperation, trust and agreement, and officials and the government are far from the people, which creates mistrust and non-acceptance of people to advice and policies of government officials including vaccinations.

In addition to these characteristics, some social characteristics also played a role in not vaccinating. Conspiracy theories and rumors about the consequences of not being vaccinated, such as sterilization and population control and reduction, were among these reasons. Consistent with our findings, Khankeh et al. (47) in a study in Iran found that tracking microchips after vaccination plays a role in preventing people from injecting it. Also, in many countries, political ideology plays a role in not vaccinating, which causes those who believe in that ideology to advocate against vaccination (9, 11, 55), indicating the importance of political leanings in vaccination. The illusion of conspiracy and rumors about the consequences of the vaccine, in addition to the political and social aspects, is rooted in religious beliefs in which religion opposes what is likely to reduce sexual potency and birth control. This ideological issue is more widespread among right-wing and conservative groups, and right-wing religious opponents of vaccination are more than non-religious ones, and this issue is also seen in Iran for religious reasons.

Social learning and observation of non-vaccination behavior in others, especially celebrities, was also a determinant of non-injection in the participants of the present study. Abramowitz et al. (56) in a study in Liberia showed that people's behavior in relation to Ebola is influenced by social learning and the behavior of other people and the media. In general, it should be said that learning is the central part of every person's life and considering that our behavior is related to the physical environment and learning from the environment, the behavior of people in society affects each other and part of the non-injection of vaccine by people of the present study can be explained by observing the behavior of other people. In this regard, influencing and teaching through famous and influential people is important.

Misconceptions about COVID-19 due to poor health knowledge were another reason not to get vaccinated. Consistent with our findings, the findings of the Ruiz and Bell study of 2020 showed that belief in immunity to a previous COVID-19 infection was one of the reasons for the reluctance to be vaccinated in the England (9). Cahapay (45) in a study among Philippines teachers found that people who do not know enough about the vaccine refuse to inject it. Various studies have referred to the negative impact of social media as one of the barriers to the Covid-19 vaccination. Also, a lot of fake news about the Covid-19 origin and treatment, as well as the false vaccination side effects, were spread through these media, which could lead to a change in people's awareness and behavior and their unwillingness to be vaccinated against the virus (36, 37). One of the misconceptions in our study was the lack of belief in the necessity of injection for various reasons, which, similar to our results, various studies have shown that the biggest obstacle to vaccination was the lack of belief in its necessity (27, 47, 49, 57). On the other hand, Sherman et al. (48) showed that the intention to be vaccinated is associated with more positive beliefs and attitudes toward COVID-19 vaccination (48). That is, having a positive belief and attitude toward the vaccine and COVID-19 can encourage a person to inject it, but the less knowledge a person has in this area, the different his attitude will be and therefore the lower the probability of injection. Health knowledge about COVID-19, vaccines in particular, due to their novelty and information about them from various and sometimes contradictory sources, promote false beliefs about the vaccine, which results in public skepticism and refusal to inject it. Providing an integrated approach to the introduction and presentation of information is a fundamental action in this regard.

The last social determinant of not being vaccinated was the fatalism due to believing in God and the other world. Consistent with our results, some studies have reported that it is possible that religiosity affects the intent or behavior of COVID-19 vaccination (6, 58). Fatalism in Iran is one of the main challenges for health professionals to intervene. Fatalism is common in religious societies, rooted in the eternal power of the deity and that everything depends on His will, and this does not make following the hygienic instructions and confronting COVID-19 a behavioral priority. Fatalism and non-injection of COVID-19 vaccine in Iranian society, which is a religious and traditional society, deserves special attention and appropriate measures.

Large-scale legal and managerial determinants also played a role in preventing vaccination. Incomplete, ambiguity and contradiction information about COVID-19 disease and available vaccines and the need for injecting vaccines was one of them. Information and knowledge from the distant past have always played a role in the tendency to inject vaccines. Liem et al. (59) stated one of the reasons for not following the health tips and not protecting themselves against COVID-19 as the lack of accurate information and the lack of understanding of the danger of the disease. However, a study by Sherman et al. (48) in England, showed that having more sufficiently understood information to make an informed decision about COVID-19 vaccination is effective in injecting it. Regarding incomplete and contradictory information, it's worth noting that in times of public crisis, governments must quickly and efficiently communicate crisis information effectively and efficiently to members of the community, as failure to do so will inevitably lead citizens to fear, uncertainty and distrust, and failure to follow instructions and anxiety which regarding vaccination, leads to a lack of willingness to be vaccinated and the persistence of the disease in the community.

Difficult and erratic access to vaccination centers was another reason for not receiving vaccines. Numerous studies have examined the issue of access and its impact on community health (47, 60, 61), because access to health services is one of the main goals of policy makers and an appropriate strategy to achieve justice in health and disease control. Therefore, access to vaccines in the shortest possible time is one of the main priorities to encourage and increase vaccination, because in the current situation in Iran, despite major problems and challenges, especially in the economic field, high expectations for vaccines and crowded centers, reduces people's desire for referral.

Lack of restrictions and compulsion to vaccinate, especially in the early days of general vaccination, also contributed to the lack of vaccination. Another studies have shown similar result (31, 62). In Iran, at the beginning of the vaccination, there was no compulsion or restrictions for non-vaccinated people, and opponents of the vaccine did not see any obstacles in their way, but over time restrictions were imposed and non-vaccinated people were barred from receiving certain services or entering certain places, but these measures lacked executive and legal support and are mostly communicated within the framework of general instructions, but they are not implemented.

Lack of incentives to vaccinate is the last determinant of non-injection of the vaccine in the study participants. Consistent with our results, studies have shown, receiving a reward was a major factor in the willingness to receive the vaccine (23, 63). Using incentives to encourage healthy behaviors, such as vaccinations, can be motivating and can be a ground for building collective safety and reducing the prevalence of the virus and the resulting deaths.

## Limitations and strengths

This research is one of the first researches that seeks to identify the determinants of non-vaccination in the city of Urmia in Iran with a qualitative method that can provide new and first-class information to health and social planners so that they can increase people's intention to inject vaccine. Qualitative studies can provide psychological details not revealed by quantitative approaches.

There were some limitations in this study. One of the main limitations was that some participants feared being questioned later for not being vaccinated. Therefore, they did not want to participate in the research, but the researchers satisfied them by explaining the conditions of the interview and participation in the research, as well as explaining that their names and addresses will remain confidential during the publication of the findings. The dissatisfaction of some participants with the recording of the interview was another limitation of the research, which forced the researchers to exclude 5 participants from the research, while these 5 people could provide useful information. Another limitation was that qualitative analyses have a low degree of generalizability.

## Conclusion

The results showed that various determinants at individual, socio-cultural and legal-managerial levels were involved in the non-injection of COVID-19 vaccine. Efforts to increase vaccination coverage require comprehensive action at various levels and the interactive cooperation of governmental and non-governmental institutions and organizations. In this regard, increase information and knowledge and exposure to social media, creating advertising and training campaigns on the safety and effectiveness of COVID-19 vaccines, using community leaders or specialists and celebrities and vaccinated people as vaccination preachers to influence the population seems useful. Also, emphasis on religious teachings related to the need to prevent and avert potential danger, improving accessibility by increasing the number of vaccination locations to reduce travel time and avoid wastage of time, rebuilding public confidence in government, using communication strategies and scientific evidence to address concerns of opponents of the vaccine and the use of a system of encouragement and punishment are also recommended to increase the willingness and action of the people to vaccinate COVID-19.

## Data Availability Statement

The raw data supporting the conclusions of this article will be made available by the authors, without undue reservation.

## Ethics Statement

The study was provided ethical approval by the Urmia University of Medical Sciences (IR.UMSU.REC.1400.430). Written consent was obtained from all participants. All procedures performed in studies involving human participants were in accordance with the ethical standards of the institutional and/or national research committee and with the 1964 Helsinki declaration and its later amendments or comparable ethical standards.

## Author contributions

JYL, SFI, SS, SA, and RH participated and approved the study design. JYL, RH, and SFI contributed to designing the study. SS collected the data. SFI, SA, and JYL analyzed the data. SFI, JYL, and RH wrote the final article. All authors read and approved the final manuscript.

## Conflict of interest

The authors declare that the research was conducted in the absence of any commercial or financial relationships that could be construed as a potential conflict of interest.

## Publisher's note

All claims expressed in this article are solely those of the authors and do not necessarily represent those of their affiliated organizations, or those of the publisher, the editors and the reviewers. Any product that may be evaluated in this article, or claim that may be made by its manufacturer, is not guaranteed or endorsed by the publisher.
